# Physical activity but not sedentary activity is reduced in primary Sjögren’s syndrome

**DOI:** 10.1007/s00296-016-3637-6

**Published:** 2016-12-24

**Authors:** Wan-Fai Ng, Ariana Miller, Simon J. Bowman, Elizabeth J. Price, George D. Kitas, Colin Pease, Paul Emery, Peter Lanyon, John Hunter, Monica Gupta, Ian Giles, David Isenberg, John McLaren, Marian Regan, Annie Cooper, Steven A. Young-Min, Neil McHugh, Saravanan Vadivelu, Robert J. Moots, David Coady, Kirsten MacKay, Bhaskar Dasgupta, Nurhan Sutcliffe, Michele Bombardieri, Costantino Pitzalis, Bridget Griffiths, Sheryl Mitchell, Samira Tatiyama Miyamoto, Michael Trenell, on behalf of the UK Primary Sjögren’s Syndrome Registry, Frances Hall, Elalaine C. Bacabac, Robert Moots, Kuntal Chakravarty, Shamin Lamabadusuriya, Michele Bombardieri, Constantino Pitzalis, Nurhan Sutcliffe, Nagui Gendi, Rashidat Adeniba, John Hamburger, Andrea Richards, Saaeha Rauz, Sue Brailsford, Joanne Logan, Diarmuid Mulherin, Jacqueline Andrews, Paul Emery, Alison McManus, Colin Pease, Alison Booth, Marian Regan, Theodoros Dimitroulas, Lucy Kadiki, Daljit Kaur, George Kitas, Mark Lloyd, Lisa Moore, Esther Gordon, Cathy Lawson, Monica Gupta, John Hunter, Lesley Stirton, Gill Ortiz, Elizabeth Price, Gavin Clunie, Ginny Rose, Sue Cuckow, Susan Knight, Deborah Symmons, Beverley Jones, Shereen Al-Ali, Andrew Carr, Katherine Collins, Ian Corbett, Christine Downie, Suzanne Edgar, Marco Carrozzo, Francisco Figuereido, Heather Foggo, Katherine James, Dennis Lendrem, Iain Macleod, Philip Mawson, Sheryl Mitchell, Andini Natasari, Philip Stocks, Jessica Tarn, Adrian Jones, Peter Lanyon, Alice Muir, Paula White, Steven Young-Min, Susan Pugmire, Saravanan Vadivelu, Annie Cooper, Marianne Watkins, Anne Field, Stephen Kaye, Devesh Mewar, Patricia Medcalf, Pamela Tomlinson, Debbie Whiteside, Neil McHugh, John Pauling, Julie James, Nike Olaitan, Mohammed Akil, Jayne McDermott, Olivia Godia, David Coady, Elizabeth Kidd, Lynne Palmer, Bhaskar Dasgupta, Victoria Katsande, Pamela Long, Charles Li, Usha Chandra, Kirsten MacKay, Stefano Fedele, Ada Ferenkeh-Koroma, Ian Giles, David Isenberg, Helena Maconnell, Stephen Porter, Paul Allcoat, John McLaren

**Affiliations:** 1 Musculoskeletal Research Group, Institute of Cellular Medicine and NIHR Biomedical Research Centre for Ageing and Chronic Disease, Newcastle University, Newcastle upon Tyne, UK; 2 MoveLab, Physical Activity and Exercise Research, Institute of Cellular Medicine and NIHR Biomedical Research Centre for Ageing and Chronic Disease, Newcastle University, Newcastle upon Tyne, UK; 3 University Hospital Birmingham, Birmingham, UK; 4 Great Western Hospitals NHS Foundation Trust, Swindon, UK; 5 Department of Rheumatology, Dudley Group of Hospitals NHS Trust, Dudley, UK; 6 Institute of Rheumatic and Musculoskeletal Medicine, NIHR Leeds Musculoskeletal Biomedical Research Unit, Leeds Teaching Hospitals Trust, University of Leeds, Leeds, UK; 7 Nottingham University Hospital, Nottingham, UK; 8 Gartnavel General Hospital, Glasgow, UK; 9 University College London Hospitals NHS Foundation Trust, London, UK; 10 NHS Fife, Whyteman’s Brae Hospital, Kirkcaldy, UK; 11 Royal Derby Hospital, Derby, UK; 12 Royal Hampshire County Hospital, Winchester, UK; 13 Portsmouth Hospitals NHS Trust, Portsmouth, UK; 14 Royal National Hospital for Rheumatic Diseases, Bath, UK; 15 Queen Elizabeth Hospital, Gateshead, UK; 16 Aintree University Hospitals, Liverpool, UK; 17 Royal Sunderland Hospital, Sunderland, UK; 18 Torbay Hospital, Torquay, UK; 19 Southend University Hospital, Westcliff-on-Sea, UK; 20 Barts and the London School of Medicine and Dentistry, London, UK; 21 Newcastle upon Tyne Hospitals NHS Foundation Trust, Newcastle upon Tyne, UK; 22 Universidade Federal do Espirito Santo, Vitoria and Universidade Federal de São Paulo, São Paulo, Brazil

**Keywords:** Primary Sjögren’s syndrome, Physical activity, Patient registry, Patient-reported outcomes, Fatigue

## Abstract

The aim of the study was to evaluate the levels of physical activity in individuals with primary Sjögren’s syndrome (PSS) and its relationship to the clinical features of PSS. To this cross-sectional study, self-reported levels of physical activity from 273 PSS patients were measured using the International Physical Activity Questionnaire-short form (IPAQ-SF) and were compared with healthy controls matched for age, sex and body mass index. Fatigue and other clinical aspects of PSS including disease status, dryness, daytime sleepiness, dysautonomia, anxiety and depression were assessed using validated tools. Individuals with PSS had significantly reduced levels of physical activity [median (interquartile range, IQR) 1572 (594–3158) versus 3708 (1732–8255) metabolic equivalent of task (MET) × min/week, *p* < 0.001], but similar levels of sedentary activity [median (IQR) min 300 (135–375) versus 343 (223–433) (MET) × min/week, *p* = 0.532] compared to healthy individuals. Differences in physical activity between PSS and controls increased at moderate [median (IQR) 0 (0–480) versus 1560 (570–3900) MET × min/week, *p* < 0.001] and vigorous intensities [median (IQR) 0 (0–480) versus 480 (0–1920) MET × min/week, *p* < 0.001]. Correlation analysis revealed a significant association between physical activity and fatigue, orthostatic intolerance, depressive symptoms and quality of life. Sedentary activity did not correlate with fatigue. Stepwise linear regression analysis identified symptoms of depression and daytime sleepiness as independent predictors of levels of physical activity. Physical activity is reduced in people with PSS and is associated with symptoms of depression and daytime sleepiness. Sedentary activity is not increased in PSS. Clinical care teams should explore the clinical utility of targeting low levels of physical activity in PSS.

**Electronic supplementary material:**

The online version of this article (doi:10.1007/s00296-016-3637-6) contains supplementary material, which is available to authorized users.

## Introduction

Musculoskeletal pain and fatigue are key symptoms of primary Sjögren’s syndrome (PSS) and can adversely impact on levels of physical activity. Indeed, individuals with PSS have reported that the disease impacts on their physical activity levels [1]. A physically active lifestyle is associated with decreased risk of cardiovascular disease, stroke, hypertension, type 2 diabetes, obesity, some cancers as well as all-cause mortality [2–4]. It is possible that people with PSS are at excess risk of secondary disease as a result of reduced physical activity in addition to the primary clinical diagnosis.

Very little research has been undertaken to evaluate physical activity levels in PSS patients. To date, a Dutch study has reported physical activity in people with PSS as its primary outcome. A report of 223 PSS patients and 67 controls revealed that PSS patients reported less moderate and vigorous physical activity, but similar levels of walking to matched controls without PSS [5]. Although an important study, the data are limited by the relatively small control group and is missing data on body mass index (BMI). Matching for BMI is important as BMI is related to physical activity [6, 7]. Another report from Sweden also suggested that physical activity is reduced in PSS, although physical activity was not the primary outcome and the study is limited by a small sample size (*n* = 51) and use of tool that is not validated [8]. Furthermore, it has been known that physical activity behaviour varies greatly across different countries in population-based studies [9, 10], and to our knowledge, physical activity in PSS has not been assessed in the UK. Interestingly, no studies have reported whether people with PSS spend more time sedentary, despite the powerful role of sedentary behaviour in health and well-being.

The International Physical Activity Questionnaire-short form (IPAQ-SF) is the most widely used self-reported questionnaire for population or cohort studies of habitual physical activities which is easy to complete, and its reliability and validity have been established in different countries [11, 12].

The primary aim of this study was to assess the level of physical activity and sedentary behaviour in a large cohort of PSS patients and people without chronic disease individually matched for age, gender and BMI. The secondary aims were to explore the relationships between physical activity and sedentary behaviour with clinical features of PSS.

## Patients and methods

To this cross-sectional study, PSS patients and healthy control subjects were recruited from September 2009 to September 2011. Self-reported levels of physical activity from PSS patients were compared with healthy controls. Fatigue and other clinical aspects of PSS including disease status, dryness, daytime sleepiness, dysautonomia, anxiety and depression were also assessed.

### Subject groups

All PSS patients are participants of the United Kingdom primary Sjögren’s syndrome registry (UKPSSR, www.sjogrensregistry.org) [13] and were recruited from 30 participating centres in the UK. Embedded within the design of the UKPSSR are several optional sub-studies aiming to address different clinical questions, one of which is the level of physical activity among PSS patients. Participants of the UKPSSR were given the IPAQ-SF questionnaire which they could choose whether to complete or not. Each PSS participant with complete IPAQ-SF data matched case by case (1:1) by sex, age (±3 years) and BMI (±3 kg/m^2^) was recruited from a community control cohort of 800 subjects without clinical diagnosis of chronic disease based on self-reported history established by co-author Trenell.

Inclusion criteria were as follows: age over 18, fulfil the American European Consensus Group (AECG) classification criteria [14] and those with complete datasets for physical activity assessment and matched controls. Patients without matched controls and body mass index (BMI) data were excluded.

Research ethical approval was granted by the North West Research Ethics Committee. Informed consent was obtained from all patients according to the principles of the Helsinki Declaration.

### Measures

All clinical and laboratory data were collected prospectively at the time of recruitment, and the instruments used were: IPAQ-SF, 100-mm visual analogue scale to determine overall fatigue, Profile of Fatigue (ProF, measures fatigue in PSS) [15], Epworth Sleepiness Scale (ESS, measures daytime sleepiness) [16], Orthostatic Grading Scale (OGS, measures orthostatic intolerance) [17], Composite Autonomic Symptom Scale (COMPASS, measures autonomic symptoms) [18], EULAR Sjögren’s Syndrome Disease Activity Index (ESSDAI, measures disease activity) [19], EULAR Sjögren’s Syndrome Patient-Reported Index (ESSPRI, measures overall burden of symptoms) [20], EULAR Sicca Score (EULAR-SS, measures overall severity of dryness) [20], Hospital Anxiety and Depression Scale (HADS, measures anxiety and depressive symptoms) [21], EuroQol-5 Domain (EQ5D, measures health-related quality of life) [22] and Comorbidity–Polypharmacy Score (CPS, quantify the magnitude of comorbid conditions) [23, 24]. To score the CPS, all the known comorbidities and medications that a patient has been taking were considered.

Physical activity was measured using IPAQ-SF. The nine-item IPAQ-SF records the time spent on physical activity of three intensity levels (vigorous, moderate and walking) as well as the time spent on sitting (referred to as sedentary in this study) in the past week. The data were processed and analysed according to the guidelines published by the IPAQ research committee [25]. In brief, the metabolic equivalent of task (MET) values for each intensity level of physical activity, derived from the IPAQ Reliability Study [12], were multiplied by the time spent (in minutes/week) for each intensity level of physical activity to obtain the total MET values for each intensity levels of physical activity. The total physical activity (PA) score is the sum of total MET values for vigorous, moderate and walking activities. Sedentary time (median) was reported in minutes/week.

### Statistical analysis

All data were analysed using IBM SPSS Statistics 19. As most variables were not normally distributed, Wilcoxon matched pairs/Mann–Whitney test was performed for comparisons between PSS and control groups. The PA scores were substantially positively skewed and were therefore log transformed. Pearson’s and Spearman’s correlations were used for correlation analysis for nonparametric and parametric data, respectively. To identify independent predictors for physical activity, stepwise linear regression analysis was performed using log-transformed total PA score as the dependent variable.

## Results

**Fig. 1:**
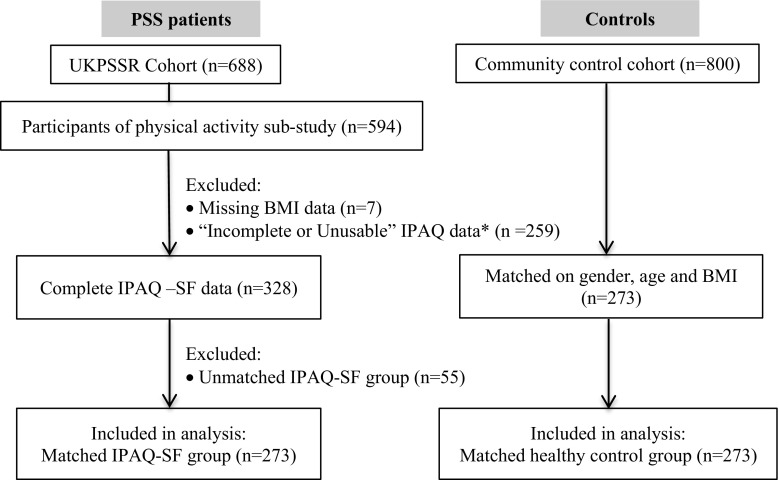
Summary of the participant flow of the study. *The majority (~70%) of the “Incomplete/unusable” data were “unusable” because the participants had responded “Don’t know/Not sure” to the question on “how much time spent on the physical activity”, the remaining were “unusable” because data on the number of days or hours/minutes spending on the physical activity were missing, unclear or contradictory

Among 688 patients recruited to the UKPSSR, 594 patients (86.3%) participated in the physical activity sub-study. Only those with complete datasets for physical activity assessment and matched controls (*n* = 273) were included in the analysis. Figure 1 summarizes the participant flow through this study.

### Patient characteristics

The clinical characteristics of the PSS cohort used in this study and the demographics of the age-, sex- and BMI-matched healthy controls are summarized in Table 1. The study cohort (“matched IPAQ-SF” group, (*n* = 273) differed clinically from the remaining UKPSSR cohort (*n* = 415), with the study cohort being younger, with better quality of life, fewer comorbidities and less overall symptom burden (ESSPRI), fatigue, pain, symptoms of anxiety, depression and daytime sleepiness (Online Resource, Supplementary table 1). There were no significant differences in the physical activity measures between the study group and those with complete IPAQ-SF datasets but without matched controls (“unmatched IPAQ-SF” group, *n* = 55), with the exception of age and BMI, which were expected because the more extreme values of the age and BMI in the unmatched IPAQ-SF group were the reasons for matching not being achieved (Online Resource, Supplementary table 2). The “matched IPAQ-SF” group also has lower CPS score than the “unmatched IPAQ-SF” group.

**Table 1:** Characteristics of the primary Sjögren’s syndrome (PSS) cohort and matched healthy control

	PSS cohort	Healthy control	*p*
Sample size	273	273	
Female/male, *n*	254/19	254/19	
Age	57 (47–65)	58 (47–65)	0.310
Body mass index (kg/m^2^)	25 (23–28)	25 (23–27)	0.285
Sitting time (min)	300 (135–375)	343 (223–433)	0.454
Vigorous PA (MET × min/wk)	0 (0–480)	480 (0–1920)	<0.001
Moderate PA (MET × min/wk)	0 (0–480)	1560 (570–3930)	<0.001
Walking (MET × min/wk)	792 (396–2079)	990 (462-3020)	0.012
Total PA score (MET × min/wk)	1572 (594–3158)	3708 (1732–8255)	<0.001

All values are presented as medians (interquartile ranges)

*kg/m* ^*2*^ kilogram per metre squared, *min* minutes, *MET* metabolic equivalent of task, *min/wk* minutes per week

### Levels of physical activity were significantly reduced in PSS patients

**Fig. 2:**
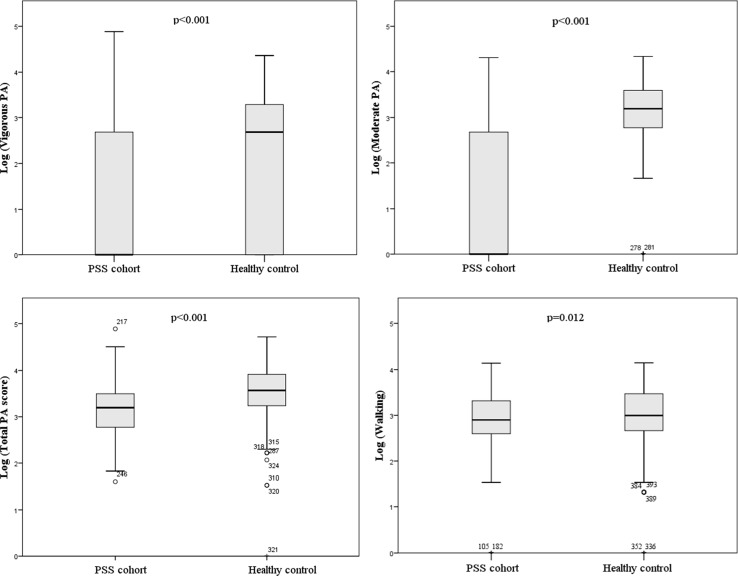
Vigorous, moderate, total and walking physical activity levels in primary Sjögren’s syndrome (PSS) cohort and healthy controls. *PA* physical activity

The median total PA score in the PSS group was <50% compared to the controls (Table 1). Moderate and vigorous physical activities were markedly reduced among PSS patients compared to the matched control group (Fig. 2). Levels of sedentary activity (sitting time) were similar between PSS patients and controls. Female patients were less active than male patients, but there was no difference in the control group (Online Resource, Supplementary table S3).

### Relationships between physical activity levels and clinical features of PSS

To explore the relationships between levels of physical activity and clinical features of PSS, we first performed a correlation analysis between total PA score and a range of pre-specified parameters based on potential biological links and data from previous studies. Total PA score correlated weakly but statistically significantly with physical fatigue (*r* = −0.159), mental fatigue (*r* = −0.135), symptoms of depression (*r* = −0.146), orthostatic intolerance (*r* = −0.124) and quality of life (*r* = −0.200), but none of the clinical features correlated with sitting time (Online Resource, Supplementary table S4). Stepwise linear regression analysis identified symptoms of depression and daytime sleepiness as independent predictors of total PA score (Table 2). However, these two predictors accounted for only approximately 4.5% of the variance in total PA score (*p* = 0.047). Fatigue was a predictor of vigorous and moderate intensities of physical activity, whereas symptoms of depression, anxiety and daytime sleepiness were predictors of physical activity of moderate intensity. Symptoms of dryness, depression and BMI predicted walking activity (Table 2).

**Table 2:** Stepwise regression analysis of independent correlations of total and various intensity levels of physical activity of primary Sjögren’s syndrome cohort

	Coefficients							
	Unstandardized		Standardized					
	B	SE	Beta	*t*	*p*	*R*	*R* ^2^	Adj. *R* ^2^
*(a) Total PA score*						0.211	0.045	0.036
(Constant)	3.191	0.075		54.148	<0.001			
Depression (HADS)	−0.031	0.010	−0.218	−3.105	0.002			
Daytime sleepiness (ESS)	0.016	0.008	0.140	2.000	0.047			
*(b) Vigorous PA*					0.308	0.095	0.087	
(Constant)	3.019	0.484		6.234	<0.001			
Physical fatigue (ProF)	−0.210	0.55	−0.244	−3.848	<0.001			
Age	−0.024	0.008	−0.189	−2.977	0.003			
*(c) Moderate PA*					0.346	0.120	0.104	
(Constant)	1.047	0.222		4.709	<0.001			
Overall fatigue (VAS)	−0.012	0.004	−0.228	−2.790	0.006			
Daytime sleepiness (ESS)	0.073	0.021	0.246	3.452	0.001			
Depression (HADS)	−0.093	0.034	−0.248	−2.781	0.006			
Anxiety (HADS)	0.086	0.027	0.264	3.209	0.002			
*(d) Walking*					0.289	0.084	0.071	
(Constant)	3.321	0.320		10.382	<0.001			
EULAR Sicca Score	0.065	0.018	0.238	3.538	<0.001			
Depression (HADS)	−0.029	0.012	−0.161	−2.390	0.018			
Body mass index	−0.028	0.012	−0.149	−2.321	0.021			

*PA* physical activity, *HADS* Hospital Anxiety and Depression Scale, *ESS* Epworth Sleepiness Scale, *ProF* Profile of Fatigue, *VAS* visual analogue scale

## Discussion

In this study, we showed that self-reported physical activity levels were significantly lower among PSS patients, particularly for physical activities of vigorous and moderate intensities, compared to age-, sex- and BMI-matched healthy controls. However, levels of sedentary activity (sitting time) were not increased in PSS patients. Reduced levels of total physical activity score were independently associated with symptoms of depression and daytime sleepiness.

Our data reveal a decrease in physical activity in people with PSS against a well-matched control group. The data build on previous reports, supporting the association of PSS with reduced moderate and vigorous physical activity [5], but add further by demonstrating that this is not confounded by BMI. Our patients were recruited from 30 centres (with a mixture of teaching hospitals and district general hospitals) across the UK, increasing the ecological validity of the data. Sedentary activity is associated with adverse health effects independent of those from decreased levels of overall physical activity or higher-intensity physical activity [26, 27]. Interestingly, the decrease in physical activity in the present study was not accompanied by an increase in sedentary behaviour. The similarity in sedentary behaviour between the PSS and control groups does not support the belief that PSS patients spend more time sitting. Combined, these data suggest that patients are moving as much as their healthy counterparts, but when they are active they are not working as hard. As such, patients with PSS may be exposed to excess risk of developing secondary chronic disease as a result of their low levels of physical activity and this should be targeted therapeutically.

Despite the weak association, our study is the first to demonstrate the relationship between physical activity and daytime sleepiness in PSS, which together with symptoms of depression, predicted lower levels of physical activities, mainly those at moderate intensity levels. In a previous study, excessive daytime sleepiness, defined as the propensity to fall asleep at a time when the individual would usually be awake and alert, was more prevalent in PSS patients than in healthy controls and was associated with mental and physical fatigue. Sleep disturbances in PSS, anxiety, nocturia and sicca problems, were also more prevalent, but only insomnia correlated with daytime sleepiness and depression had some impact on daytime sleepiness [28]. In a study by Zafar et al., PSS patients had higher level of daytime sleepiness and twice the frequency of obstructive apnoeas and hypopneas compared with control subjects but no significant correlations were found between these parameters and sleepiness scores [29]. Walker et al. [30] found that PSS patients have more severe symptoms of daytime sleepiness than patients with osteoarthritis, independent of nocturia. Daytime sleepiness in PSS is also associated with impaired functional status [31], autonomic symptoms [32] and decreased quality of life [33]. However, daytime sleepiness and depression can be interrelated and should be viewed concurrently.

Depression is a key determinant of EQ5D utility values in a large PSS cohort in the UK and also has a significant correlation with fatigue [33]. Depression is an independent predictor of cognitive symptoms [34], strongly correlates with functional disability [31] and leads to more physician visits and work disability in PSS patients [35]. Strömbeck et al. [8] did not find an association between depression and an indirect measure of aerobic capacity. In contrast, our study demonstrated that depression may be an independent predictor of decreased levels of self-reported physical activity in PSS. Our data suggest that identifying excessive daytime sleepiness and depressive symptoms as well as their contributing factors may be important in achieving a more physically active lifestyle for some people living with PSS.

The inverse relationship between fatigue and moderate/vigorous physical activity levels is perhaps unsurprising and has been reported in healthy populations and other chronic conditions [36–39]. Whether there is a causal link between fatigue and physical activity levels remains unclear and cannot be addressed based on the data generated from this study. Strömbeck et al. reported that women with PSS have decreased physical capacity and experienced more pain during the shoulder mobility test. Furthermore, diminished aerobic capacity correlated with symptoms of fatigue experienced [8]. Interestingly, in a small group of PSS patients, Nordic walking for 45 min thrice weekly for 12 weeks improved aerobic capacity and reduced symptoms of fatigue and depression, but not quality of life [40]. More recently, Wouters et al. [5] have shown that in PSS patients’ lower physical activity, higher activity avoidance and somatic focus were associated with more severe symptoms of fatigue. These observations implied that programmes designed to increase the levels of physical activity may ameliorate the symptoms of fatigue and depression.

The association between symptoms of dryness and physical activity levels is a novel finding and could be a consequence of the increased walking time compared with vigorous and moderate activities in patients with higher symptoms of dryness. Further studies are needed to explore the underlying mechanisms of association between sicca symptoms and physical activity levels.

In this study, symptoms of depression and daytime sleepiness accounted for merely 4.5% of the variance of total physical activity levels. Symptoms of pain, autonomic dysfunction and anxiety are well described among PSS patients and may also affect physical activity levels. However, although fatigue and orthostatic intolerance inversely correlated with total physical activity score, they were not identified as independent predictors in regression analysis. Given the link between reduced physical activity levels and adverse health outcomes [2–4] as well as diminished quality of life [41–43], further studies to improve our understanding of the contributing factors to reduced physical activity in PSS and to devise strategies to increase physical activity levels in PSS are warranted.

The present study has limitations. Formal sample size calculation was not performed; nevertheless, our sample is greater than the previous study [5] and is matched case by case by sex, age and BMI. Direct measurements of physical activity were not performed; however, IPAQ-SF scores have been shown to correlate well with actual physical activity levels [11, 12]. Furthermore, since physical activity levels of both study groups were estimated using the same instrument, we anticipate that our observation of reduced physical activity levels in PSS relative to their matched healthy controls remains valid upon objective measurements. Only ~40% of the entire cohort had complete and matched IPAQ-SF data for analysis, and the study cohort differed from the remaining PSS cohort—with the study cohort having better quality of life and lower levels of fatigue, disease activity, fewer comorbidities, less symptom burden, dryness, orthostatic intolerance, anxiety, symptoms of depression and being younger. Since fatigue, depression and orthostatic intolerance are inversely correlated with the levels of physical activity, we believe that the physical activity levels of PSS patients of the entire cohort might be even lower. External validation of our findings with an independent cohort has not been conducted.

In conclusion, physical activity is reduced in people with PSS without a concomitant increase in sedentary activity. Symptoms of depression and daytime sleepiness may be predictors of physical activity in PSS. Given the risks of developing secondary chronic disease as a result of low levels of physical activity, clinicians should explore the clinical utility of increasing physical activity as part of a holistic management package of PSS.

## Electronic supplementary material

Below is the link to the electronic supplementary material.

Supplementary material 1 (PDF 229 kb)

Supplementary material 2 (PDF 232 kb)

Supplementary material 3 (PDF 224 kb)

Supplementary material 4 (PDF 232 kb)
