# Supplementary material for: Physical activity but not sedentary activity is reduced in primary Sjögren’s syndrome
Source: Rheumatol Int. 2016 Dec 24;37(4):623–31. doi: 10.1007/s00296-016-3637-6 (PMC5357288; doi:10.1007/s00296-016-3637-6)
Supplement: Supplementary file 2 — Supplementary material 2 (PDF 232 kb) [file 296_2016_3637_MOESM2_ESM.pdf]

PHYSICAL ACTIVITY BUT NOT SEDENTARY ACTIVITY IS REDUCED IN PRIMARY SJÖGREN'S SYNDROME

Rheumatology International

Wan-Fai Ng[1], Ariana Miller[2], Simon J Bowman[3], Elizabeth J Price[4], George D Kitas[5], Colin Pease[6], Paul Emery[6], Peter Lanyon[7], John Hunter[8], Monica Gupta[8], Ian Giles[9], David Isenberg[9], John McLaren[10], Marian Regan[11], Annie Cooper[12,13], Steven A Young-Min[13], Neil McHugh[14], Saravanan Vadivelu[15], Robert J Moots[16], David Coady[17], Kirsten MacKay[18], Bhaskar Dasgupta[19], Nurhan Sutcliffe[20], Michele Bombardieri[20], Costantino Pitzalis[20], Bridget Griffiths[21], Sheryl Mitchell[21], Samira Tatiyama Miyamoto[22], Michael Trenell[2] on behalf of the UK primary Sjögren's syndrome registry[23]

[1]Musculoskeletal Research Group, [2]MoveLab, Physical Activity & Exercise Research, Institute of Cellular Medicine and NIHR Biomedical Research Centre for Ageing & Chronic Disease, Newcastle University, Newcastle upon Tyne, UK

[3]University Hospital Birmingham, Birmingham, UK

[4]Great Western Hospitals NHS Foundation Trust, Swindon, UK.

[5]Department of Rheumatology, Dudley Group of Hospitals NHS Trust, Dudley, UK.

[6]Institute of Rheumatic and Musculoskeletal Medicine, University of Leeds & NIHR Leeds Musculoskeletal Biomedical Research Unit, Leeds Teaching Hospitals Trust, Leeds, UK.

[7]Nottingham University Hospital, Nottingham, UK

[8]Gartnavel General Hospital, Glasgow, UK.

[9]University College London Hospitals NHS Foundation Trust, London, UK.

[10]NHS Fife, Whyteman's Brae Hospital, Kirkcaldy, UK

[11]Royal Derby Hospital, Derby, UK.

[12]Royal Hampshire County Hospital, Winchester, UK.

[13]Portsmouth Hospitals NHS Trust, Portsmouth, UK.

[14]Royal National Hospital for Rheumatic Diseases, Bath, UK

[15]Queen Elizabeth Hospital, Gateshead, UK.

[16]Aintree University Hospitals, Liverpool, UK

[17]Royal Sunderland Hospital, Sunderland, UK

[18]Torbay Hospital, Torquay, UK

19Southend University Hospital, Westcliff-on-Sea, UK.

[20]Barts and the London School of Medicine and Dentistry, UK

[21]Newcastle upon Tyne Hospitals NHS Foundation Trust, Newcastle upon Tyne, UK.

[22] Universidade Federal do Espirito Santo, Vitoria, Universidade Federal de São Paulo, São Paulo, BR. CAPES Foundation scholar – Proc. n. BEX 8831/14-9

[23]Denotes corporate authorship. See appendix 1 for the full list of members.

Corresponding author: Wan-Fai Ng.

e-mail: Wan-fai.Ng@ncl.ac.uk

Supplementary table S2. Comparison of the primary Sjögren's syndrome cohort (matched IPAQ-SF) and unmatched IPAQ-SF cohort

	Matched IPAQ-SF group (PSS cohort)	Unmatched IPAQ-SF group	p
Sample size	273	55	
Age (years)	57 (47-65)	68 (60-73)	<0.001
Gender (Number of females, (%))	254 (93%)	48 (87%)	0.073*
Body mass index (Kg/m^2)	25 (23-28)	32 (24-37)	<0.001
Disease duration (years)	5 (2-10)	4 (2-10)	0.278
CPS	7 (5-11)	9 (7-13)	0.001
Anti-Ro/La positive (number, (%))	242 (88.6%)	46 (84%)	0.099
ESSDAI (0-123)	3 (1-7)	4 (1-7)	0.300
ESSPRI (0-10)	5.3 (3.3-6.7)	5.3 (2.3-7.3)	0.936
EULAR-Sicca Score (0-10)	5.7 (3.7-7.7)	7 (3.3-8.7)	0.533
Overall fatigue (VAS, 0-100)	55 (29-72)	67 (22-76)	0.487
Physical fatigue (ProF, 0-7)	3.5 (2.3-4.8)	3.8 (1.9-5.3)	0.843
Mental fatigue (ProF, 0-7)	2.5 (1-4)	2.5 (1-4)	0.508
Pain (0-10)	4 (1-6.8)	4 (2-7)	0.669
Anxiety (HADS, 0-21)	7 (4-10)	6.5 (3-10)	0.374
Depression (HADS, 0-21)	4 (2-7)	6 (3-9)	0.128
Daytime sleepiness (ESS, 0-24)	7 (4-11)	8 (4-11)	0.566
Dysautonomia - COMPASS (0-170)	39 (27-50)	39 (23-54)	0.639
OGS (5-25)	3 (0-5)	2 (0-3)	0.031
EQ-5D - TTO (-1 to 1)	0.73 (0.66-0.80)	0.73 (0.62-0.80)	0.258
VAS (0 – 100)	70 (50-80)	70 (40-80)	0.617
Physical Activity Measures			
Sitting time (min)	300 (135-375)	270 (180-420)	0.328
Moderate PA (MET*min/week)	0 (0-480)	0 (0-400)	0.265
Vigorous PA (MET*min/week)	0 (0-480)	0 (0-0)	0.465
Walking (MET*min/week)	792 (396-2079)	594 (248-2376)	0.344
Total PA score (MET*min/week)	1572 (594-3158)	1386 (396-4692)	0.556

All values are presented as medians (interquartile ranges) with the exception of sample size gender and anti-Ro/La positivity. *Fisher exact test.

IPAQ-SF: International Physical Activity Questionnaire – short form; kg/m^2: kilogram-meter squared; CPS: Comorbidity-Polypharmacy score; ESSDAI: EULAR Sjögren Syndrome Disease Activity Index; ESSPRI: EULAR Sjögren Syndrome Patient Reported Index; VAS: visual analogue scale; ProF: Profile of Fatigue ; HADS: Hospital Anxiety and Depression Scale; ESS: Epworth Sleepiness Scale; COMPASS: Composite Autonomic Symptom Scale; OGS: Orthostatic Grading Scale; EQ-5D: EuroQol 5-domain; TTO: time trade-off; PA: physical activity; min: minutes; MET: metabolic equivalent of task; min/wk: minutes per week
