# Supplementary material for: Physical activity but not sedentary activity is reduced in primary Sjögren’s syndrome
Source: Rheumatol Int. 2016 Dec 24;37(4):623–31. doi: 10.1007/s00296-016-3637-6 (PMC5357288; doi:10.1007/s00296-016-3637-6)
Supplement: Supplementary file 3 — Supplementary material 3 (PDF 224 kb) [file 296_2016_3637_MOESM3_ESM.pdf]

PHYSICAL ACTIVITY BUT NOT SEDENTARY ACTIVITY IS REDUCED IN PRIMARY SJÖGREN'S SYNDROME

Rheumatology International

Wan-Fai Ng[1], Ariana Miller[2], Simon J Bowman[3], Elizabeth J Price[4], George D Kitas[5], Colin Pease[6], Paul Emery[6], Peter Lanyon[7], John Hunter[8], Monica Gupta[8], Ian Giles[9], David Isenberg[9], John McLaren[10], Marian Regan[11], Annie Cooper[12,13], Steven A Young-Min[13], Neil McHugh[14], Saravanan Vadivelu[15], Robert J Moots[16], David Coady[17], Kirsten MacKay[18], Bhaskar Dasgupta[19], Nurhan Sutcliffe[20], Michele Bombardieri[20], Costantino Pitzalis[20], Bridget Griffiths[21], Sheryl Mitchell[21], Samira Tatiyama Miyamoto[22], Michael Trenell[2] on behalf of the UK primary Sjögren's syndrome registry[23]

[1]Musculoskeletal Research Group, [2]MoveLab, Physical Activity & Exercise Research, Institute of Cellular Medicine and NIHR Biomedical Research Centre for Ageing & Chronic Disease, Newcastle University, Newcastle upon Tyne, UK

[3]University Hospital Birmingham, Birmingham, UK

[4]Great Western Hospitals NHS Foundation Trust, Swindon, UK.

[5]Department of Rheumatology, Dudley Group of Hospitals NHS Trust, Dudley, UK.

[6]Institute of Rheumatic and Musculoskeletal Medicine, University of Leeds & NIHR Leeds Musculoskeletal Biomedical Research Unit, Leeds Teaching Hospitals Trust, Leeds, UK.

[7]Nottingham University Hospital, Nottingham, UK

[8]Gartnavel General Hospital, Glasgow, UK.

[9]University College London Hospitals NHS Foundation Trust, London, UK.

[10]NHS Fife, Whyteman's Brae Hospital, Kirkcaldy, UK

[11]Royal Derby Hospital, Derby, UK.

[12]Royal Hampshire County Hospital, Winchester, UK.

[13]Portsmouth Hospitals NHS Trust, Portsmouth, UK.

[14]Royal National Hospital for Rheumatic Diseases, Bath, UK

[15]Queen Elizabeth Hospital, Gateshead, UK.

[16]Aintree University Hospitals, Liverpool, UK

[17]Royal Sunderland Hospital, Sunderland, UK

[18]Torbay Hospital, Torquay, UK

19Southend University Hospital, Westcliff-on-Sea, UK.

[20]Barts and the London School of Medicine and Dentistry, UK

[21]Newcastle upon Tyne Hospitals NHS Foundation Trust, Newcastle upon Tyne, UK.

[22] Universidade Federal do Espirito Santo, Vitoria, Universidade Federal de São Paulo, São Paulo, BR. CAPES Foundation scholar – Proc. n. BEX 8831/14-9

[23]Denotes corporate authorship. See appendix 1 for the full list of members.

Corresponding author: Wan-Fai Ng.

e-mail: Wan-fai.Ng@ncl.ac.uk

Supplementary Table S3. Gender characteristics of the primary Sjögren's syndrome (PSS) cohort and matched healthy control

	PSS cohort			Healthy control		
	**Female**	**Male**	**p**	**Female**	**Male**	**p**
Sample size	254	19		254	19	
Age (years)	58 (47-65)	53 (46-63)	0.372	58 (47-65)	54 (46-60)	0.404
Body mass index (kg/m^2)	25 (23-28)	27 (24-29)	0.532	25 (23-27)	26 (23-28)	0.298
Sitting time (min)	240 (120-398)	300 (240-360)	0.798	343 (223-429)	394 (266-480)	0.976
Vigorous PA (MET*min/wk)	0 (0-480)	0 (0-1920)	0.022	480 (0-1920)	960 (0-2200)	0.380
Moderate PA (MET*min/wk)	0 (0-420)	160 (0-1200)	0.048	1560 (540-3900)	1740 (960-2880)	0.391
Walking (MET*min/wk)	792 (396-2079)	990 (495-2376)	0.999	990 (462-3069)	792 (198-2079)	0.459
Total PA score (MET*min/wk)	1140 (590-3012)	3150 (1224-6750)	0.003	3690 (1732-9739)	3990 (1700-6405)	0.846

All values are presented as medians (interquartile ranges)

kg/m^2: kilogram-meter squared; min: minutes; MET: metabolic equivalent of task; min/wk: minutes per week
