# Supplementary material for: Physical activity but not sedentary activity is reduced in primary Sjögren’s syndrome
Source: Rheumatol Int. 2016 Dec 24;37(4):623–31. doi: 10.1007/s00296-016-3637-6 (PMC5357288; doi:10.1007/s00296-016-3637-6)
Supplement: Supplementary file 4 — Supplementary material 4 (PDF 232 kb) [file 296_2016_3637_MOESM4_ESM.pdf]

PHYSICAL ACTIVITY BUT NOT SEDENTARY ACTIVITY IS REDUCED IN PRIMARY SJÖGREN'S SYNDROME

Rheumatology International

Wan-Fai Ng[1], Ariana Miller[2], Simon J Bowman[3], Elizabeth J Price[4], George D Kitas[5], Colin Pease[6], Paul Emery[6], Peter Lanyon[7], John Hunter[8], Monica Gupta[8], Ian Giles[9], David Isenberg[9], John McLaren[10], Marian Regan[11], Annie Cooper[12,13], Steven A Young-Min[13], Neil McHugh[14], Saravanan Vadivelu[15], Robert J Moots[16], David Coady[17], Kirsten MacKay[18], Bhaskar Dasgupta[19], Nurhan Sutcliffe[20], Michele Bombardieri[20], Costantino Pitzalis[20], Bridget Griffiths[21], Sheryl Mitchell[21], Samira Tatiyama Miyamoto[22], Michael Trenell[2] on behalf of the UK primary Sjögren's syndrome registry[23]

[1]Musculoskeletal Research Group, [2]MoveLab, Physical Activity & Exercise Research, Institute of Cellular Medicine and NIHR Biomedical Research Centre for Ageing & Chronic Disease, Newcastle University, Newcastle upon Tyne, UK

[3]University Hospital Birmingham, Birmingham, UK

[4]Great Western Hospitals NHS Foundation Trust, Swindon, UK.

[5]Department of Rheumatology, Dudley Group of Hospitals NHS Trust, Dudley, UK.

[6]Institute of Rheumatic and Musculoskeletal Medicine, University of Leeds & NIHR Leeds Musculoskeletal Biomedical Research Unit, Leeds Teaching Hospitals Trust, Leeds, UK.

[7]Nottingham University Hospital, Nottingham, UK

[8]Gartnavel General Hospital, Glasgow, UK.

[9]University College London Hospitals NHS Foundation Trust, London, UK.

[10]NHS Fife, Whyteman's Brae Hospital, Kirkcaldy, UK

[11]Royal Derby Hospital, Derby, UK.

[12]Royal Hampshire County Hospital, Winchester, UK.

[13]Portsmouth Hospitals NHS Trust, Portsmouth, UK.

[14]Royal National Hospital for Rheumatic Diseases, Bath, UK

[15]Queen Elizabeth Hospital, Gateshead, UK.

[16]Aintree University Hospitals, Liverpool, UK

[17]Royal Sunderland Hospital, Sunderland, UK

[18]Torbay Hospital, Torquay, UK

19Southend University Hospital, Westcliff-on-Sea, UK.

[20]Barts and the London School of Medicine and Dentistry, UK

[21]Newcastle upon Tyne Hospitals NHS Foundation Trust, Newcastle upon Tyne, UK.

[22] Universidade Federal do Espirito Santo, Vitoria, Universidade Federal de São Paulo, São Paulo, BR. CAPES Foundation scholar – Proc. n. BEX 8831/14-9

[23]Denotes corporate authorship. See appendix 1 for the full list of members.

Corresponding author: Wan-Fai Ng.

e-mail: Wan-fai.Ng@ncl.ac.uk

Supplementary table S4. Bivariate correlation analysis between IPAQ-SF and clinical features of primary Sjögren's syndrome cohort

	Total PA score	Sitting time	Moderate PA	Vigorous PA	Walking
Age	-0.048	-0.078	-0.113	-0.196**	0.065
Body mass index	-0.008	0.066	-0.004	0.030	-0.037*
Disease duration	-0.079	0.069	-0.030	-0.129*	-0.012
CPS	-0.101	0.102	-0.141*	-0.138*	-0.025
Anti-Ro/La status	-0.008	-0.046	-0.066	-0.014	-0.090
ESSDAI	-0.011	0.015	-0.091	0.049	-0.026
ESSPRI	-0.049	-0.001	-0.114	-0.190**	0.088
EULAR-Sicca Score	0.073	0.014	-0.065	-0.130*	0.201**
Overall fatigue (VAS)	-0.137*	0.025	-0.128*	-0.200**	-0.004
Physical fatigue (ProF)	-0.159**	0.048	-0.113	-0.249**	-0.033
Mental fatigue (ProF)	-0.135*	-0.029	-0.068	-0.144*	-0.063
Pain	-0.049	-0.69	-0.082	-0.168**	0.028
Anxiety (HADS)	-0.05	-0.053	0.081	-0.041	-0.051
Depression (HADS)	-0.146*	-0.007	-0.103	-0.084	-0.087
Daytime sleepiness (ESS)	0.018	0.009	-0.099	-0.017	0.012
Dysautonomia - COMPASS	-0.056	-0.113	-0.071	-0.098	0.024
OGS	-0.124*	0.085	-0.081	-0.107	-0.052
EQ-5D - TTO	0.200**	0.047	0.122*	0.175**	0.080
VAS	0.163**	-0.058	0.085	0.129*	0.100

*p<0.05, **p<0.01

IPAQ-SF: International Physical Activity Questionnaire – short form; PA: physical activity; CPS: Comorbidity-Polypharmacy score; ESSDAI: EULAR Sjögren Syndrome Disease Activity Index; ESSPRI: EULAR Sjögren Syndrome Patient Reported Index; VAS: visual analogue scale; ProF: Profile of Fatigue ; HADS: Hospital Anxiety and Depression Scale; ESS: Epworth Sleepiness Scale; COMPASS: Composite Autonomic Symptom Scale; OGS: Orthostatic Grading Scale; EQ-5D: EuroQol 5-domain; TTO: time trade-off
